# The Genetic and Clinical Outcomes in Fetuses With Isolated Fetal Growth Restriction: A Chinese Single-Center Retrospective Study

**DOI:** 10.3389/fgene.2022.856522

**Published:** 2022-04-28

**Authors:** Hang Zhou, Ken Cheng, Yingsi Li, Fang Fu, Ru Li, Yongling Zhang, Xin Yang, Xiangyi Jing, Fucheng Li, Jin Han, Min Pan, Li Zhen, Dongzhi Li, Can Liao

**Affiliations:** ^1^ Prenatal Diagnostic Center, Guangzhou Women and Children’s Medical Center, Guangzhou Medical University, Guangzhou, China; ^2^ School of Medicine, South China University of Technology, Guangzhou, China

**Keywords:** isolated fetal growth restriction, chromosomal microarray, copy number variants, prenatal diagnosis, perinatal outcome

## Abstract

**Objective:** To evaluate the utility of a chromosomal microarray (CMA) in fetuses with isolated fetal growth restriction (FGR) and explore risk factors for the prediction of chromosomal aberration and perinatal adverse outcomes.

**Method:** This study included 271 fetuses of estimated fetal weight less than the 3rd percentile without other structural malformation. Early-onset and late-onset FGR were defined as gestational weeks less than 32 weeks and more than 32 weeks respectively. These patients underwent quantitative fluorescent polymerase chain reaction (QF-PCR) and CMA as the first-line genetic detection strategy. Chromosomal anomalies were compared after stratified analysis by the early-onset and the late-onset FGR, including the absence or presence of ultrasound soft markers, abnormal amniotic fluid, abnormal umbilical Doppler, and gestational disorders. The follow-up time was within 1 year after birth. Logistic regression was used to seek risk predictors of chromosomal aberration and perinatal adverse outcomes for isolated FGR.

**Results:** The CMA identified clinically significant variants in 18/271 (6.6%) fetuses, and variants of unknown significance (VOUS) in 15/271 (5.5%) fetuses. Stratified analysis showed that there was a higher incidence of clinically significant variants in fetuses with the early-onset FGR compared with late-onset FGR (8.7%, 17/195 vs. 1.3%, 1/76, *p* < 0.05). Regression analysis showed that early gestational age (GA) at diagnosis of FGR was the major risk factor for chromosomal aberration (OR = 0.846). By variable regression analysis, early GA at diagnosis and decreased estimated fetal weight (EFW) percentile of suspicion of FGR, asymmetrical FGR, abnormal amniotic fluid, and severe preeclampsia could all increase the risk of adverse outcomes of isolated FGR including intra-uterine fetal death (IUFD), termination of pregnancy (TOP), and preterm birth in pregnancies with FGR.

**Conclusion:** This study emphasized the value of microarrays for unbalanced genomic variants in fetuses with isolated FGR, especially since the gestational age of nullipara was less than 32 weeks. Perinatal adverse outcomes of isolated FGR were influenced by multiple factors including GA and estimated fetal weight (EFW) percentile of suspicion of FGR, asymmetrical FGR, abnormal amniotic fluid, and severe preeclampsia.

## Introduction

The utility of a chromosomal microarray (CMA) for ultrasound structural defects has been well researched and recommended (South et al., 2013; Armour et al., 2018; Committee For Birth Defect Prevention And Control Chinese Association Of Preventive Medicine Genetic Testing And Precision Medicine Branch Chinese Association Of Birth Health et al., 2020). Its detection rate of pathogenic copy number variants (pCNVs) was found to be approximately 6.0% in fetuses with ultrasound structural malformation (Wapner et al., 2012). Fetal growth restriction (FGR) refers to a fetus that failed to reach its growth potential (Martins et al., 2020). However, the value of chromosomal microarray analysis for isolated FGR is yet to be completely clarified. There is also a lack of guidelines for clinical practice in the application of CMA for the indication of isolated FGR. One meta-study of chromosomal aberrations in apparently isolated intrauterine growth restriction showed that the detection rate of CMA for isolated FGR was 6.4% of the mean rate of chromosomal aberrations, from 0 to 26.3% (Sagi-Dain et al., 2017). One of reasons for the great difference in the detection rate of CMA was due to various inclusion standards for FGR by using different percentile thresholds. Some reports defined it as estimated fetal weight (EFW) was less than the 10th percentile. Other researchers recommended using a customized EFW or using a population reference for EFW (Lausman and Kingdom, 2013; Vayssière et al., 2015; Blue et al., 2018; ACOG, 2019). However, FGR was redefined for the smallest fetuses with an EFW below the 3rd percentile by a recent expert consensus (Gordijn et al., 2016).

There have been few studies about what factors would influence the detection rate of CMA in fetuses with FGR. Since the commercial explosion of CMA, most studies of CMA in FGR focused on the incremental yield compared karyotype (Borrell et al., 2018), as well as compared the detection rate of CMA between isolated FGR and FGR combined with other structural abnormalities (Zhu et al., 2016; Borrell et al., 2017; Brun et al., 2018). There were only a few studies on isolated FGR. Moreover, the difference between early-onset and late-onset FGR was described in several studies. But this led to very different conclusions due to various definitions of early-onset and late-onset FGR. Some researches demonstrated that there was a similar detection rate of CMA between the early-onset and the late-onset FGR (An et al., 2018). Some researchers suggested that the incidence of chromosomal anomalies became higher when fetuses were diagnosed with the early-onset form than the late-onset form (Peng et al., 2017; Monier et al., 2021). In addition, FGR pregnancies associated with definite causes for impaired fetal growth, such as gestational hypertension and severe preeclampsia, might be associated with a lower risk of chromosomal anomalies. But few studies of FGR have covered these vital issues.

This study aims to access CMA in fetuses with isolated FGR with EFW < 3% and to explore risk factors for the prediction of chromosomal aberration and perinatal adverse outcome.

## Materials and Methods

This retrospective study was performed in Guangzhou Women and Children’s Medical Center from January 2016 to December 2020. The research was approved by the ethics committee of Guangzhou Women and Children’s Medical Center. Data were retrieved from our medical record database. Inclusion criteria standards were as follows: (I) Fetuses were diagnosed with FGR with an EFW below the third percentile (3%). (II) Singleton pregnancy. (III) Reliable gestational ages from maternal menstrual history and first-trimester ultrasound results should be required to access gestational age. (IV) Available invasive chromosomal testing. Exclusion criteria were multiple pregnancies, diagnosis with ultrasound structural malformation but not ultrasound soft markers, as well as suspicion of TORCH (toxoplasma, rubella virus, cytomegalovirus, herpes simplex virus, others) infection.

Maternal and fetal clinical characteristics and perinatal outcome were reviewed in our medical record system, including maternal age, reproductive history, pregnant disease, GA at the suspicion of FGR and invasive procedure, outcome of pregnancy, mode of delivery, gestational age at birth, neonatal sex, birth weight, and birth weight percentile. Information such as pregnancy outcome was obtained through telephone follow-up. The follow-up time was within 1 year after birth.

The definition of FGR was set as the EFW below the third percentile (3%) by the formula of Hadlock C (Hadlock et al., 1985). Fetuses were diagnosed with asymmetrical FGR if the head to abdomen circumference (HC/AC) was above the 95th percentile for gestational age (Campbell and Thoms, 1977). Abnormal umbilical Doppler was defined as absent or reversed end-diastolic flow in the umbilical artery. Oligohydramnios and polyhydramnios were diagnosed when the largest vertical pocket measured ≤ 2 cm and the largest vertical pocket measured > 24 cm, respectively. The pregnant and neonatal outcomes were retrieved in our obstetrical and neonatal medical records. Adverse outcomes were defined as the termination of pregnancy, preterm birth, intrauterine fetal demise, and postnatal malformation.

Informed consent was obtained from the pregnant women before the invasive procedure. CMA was performed by using an Affymetrix CytoScan HD/750K array with a single-nucleotide polymorphism array (SNP array) and array-based comparative genomic hybridization (aCGH) platforms at resolutions of 10 and 100 kb respectively according to the manufacturer’s protocol (Affymetrix Inc., Santa Clara, CA, United States). The built reference genome was aligned on GRCh37/hg19. Fetal DNA was extracted from amniocytes, umbilical blood, or other family members’ peripheral lymphocytes by using a Qiagen DNA Blood Midi/Mini Kit (Qiagen GmbH, Hilden, Germany). Invasive samples were analyzed with quantitative fluorescent polymerase chain reaction (QF-PCR) by utilizing a multiplex ligation-dependent probe amplification (MLPA) kit for aneuploidy screening for chromosomes 13, 18, 21, X, and Y (Guangzhou Darui Biotechnology Co., Ltd, Guangdong, China), to exclude chromosomes 13, 18, 21, X, and Y and maternal cell contamination. Samples were subsequently subjected to CMA only when there was a normal QF-PCR result. Classification of CNVs was according to joint consensus recommendations of the American College of Medical Genetics and Genomics and ClinGen (Kearney et al., 2011; Riggs et al., 2020). The description of genomic findings identified by CMA was referred by the International System for Human Cytogenomic Nomenclature (ISCN 2020) (McGowan-Jordan Jean, 2020). The pathogenic CNVs, likely pathogenic CNVs, and variants of unknown significance (VOUS) are recorded and documented, but likely benign and benign VOUS are not considered. If a clinically significant variation or VOUS was identified in samples from the invasive procedure, parental CMA was recommended for these couples.

Statistical analysis was performed by using IBM statistical program SPSS 25.0. The Chi-square test or Fisher exact test was used for categorical data. Logistic regression analysis was performed to explore the predictors for chromosomal aberration and adverse outcome, and variables included maternal age, gestational age, GA at the suspicion of FGR, EFW at the suspicion of FGR, ultrasound soft markers, abnormal umbilical Doppler, abnormal amniotic fluid, and severe preeclampsia. A *p*-value of less than 0.05 was considered statistically significant.

## Results

**FIGURE 1:**
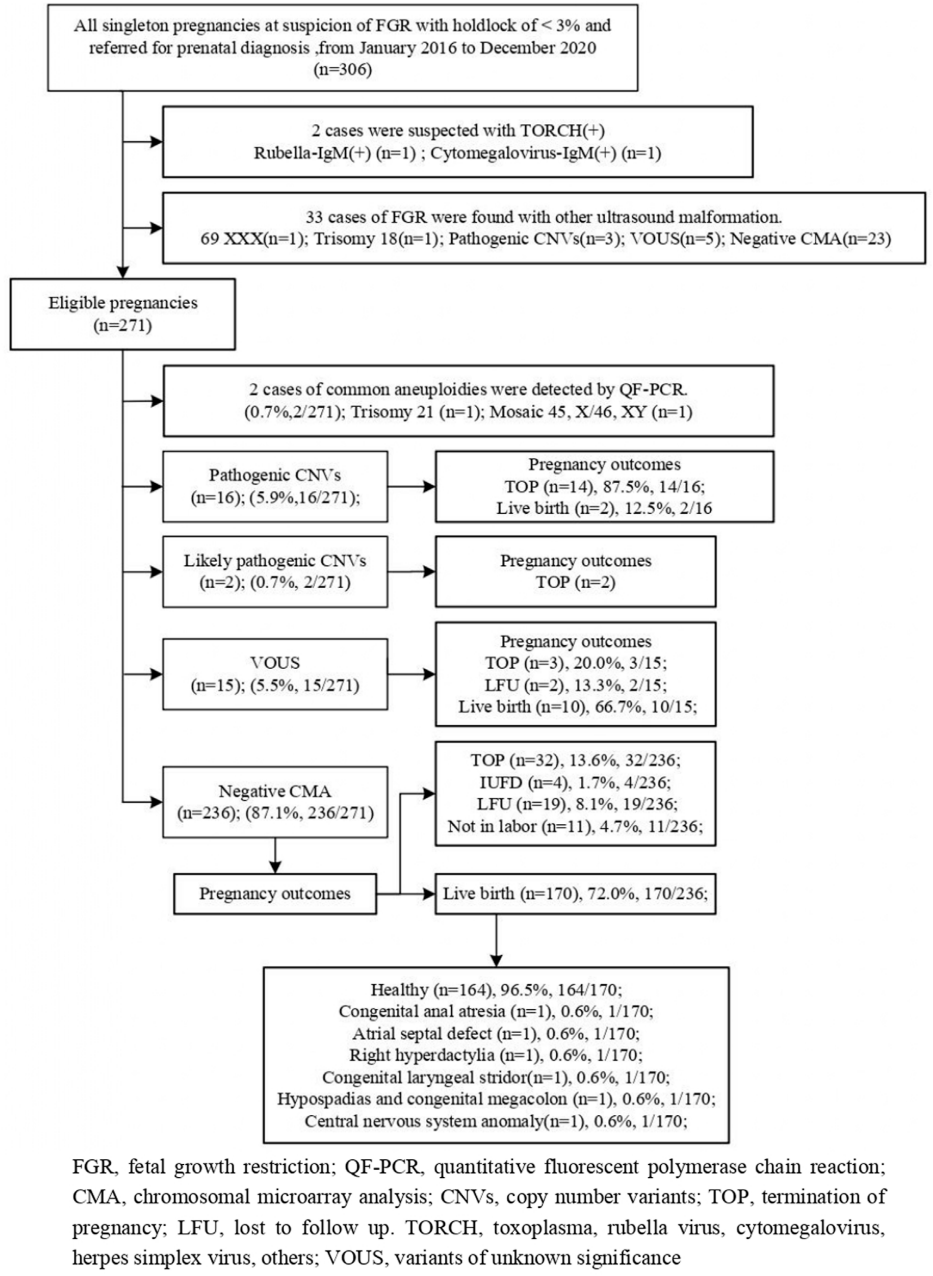
Flowchart of this study.

A total of 306 cases with the Hadlock definition of below the 3rd percentile underwent an invasive procedure from January 2016 to December 2020 in our center. There were two cases of exclusion consisting of one case with suspicion of rubella virus and one with cytomegalovirus. Overall, 33 FGR pregnancies were excluded due to fetuses with other ultrasound malformations. This study included a total of 271 cases diagnosed with isolated FGR. Amniocentesis was performed in 201 cases (74.1%) and percutaneous umbilical blood sampling was conducted in 70 cases (25.9%). There were two chromosomal numerical anomalies detected by QF-PCR including one case with abnormal sex chromosome XXY, and another one with trisomy 21 confirmed by CMA. The chromosomal microarray identified clinically significant variants in 18/271 (6.6%) including pathogenic copy number variants (pCNVs) in 16/271 (5.9%) fetuses and likely pathogenic CNVs in 2/271 (0.7%) fetuses. VOUS were found in 15/271 (5.5%) fetuses. Considering economic factors, a few couples with CNVs identified in fetuses refused the proposal to perform a parental CMA test because its price is nearly 1,000 dollars. Detailed information on prenatal outcomes and follow-up is documented in the flowchart in Figure 1.

Maternal age was 30.2 ± 4.2 years old and gestational age (GA) at diagnosis of FGR was 29.2 ± 3.8 weeks. A total of 44 (16.2%) of all cases were diagnosed with ultrasound soft markers. Among all cases, 182 (67.2%) were live births, 53 (19.6%) selected to terminate the pregnancy, and 4 (1.5%) were diagnosed with spontaneous intrauterine fetal death.

By stratified statistical analysis (see Table 1), the detection rate of clinically significant variants (8.7%, 17/195 vs. 1.3%, 1/76 *p* < 0.05) in the early-onset FGR group was higher, but the rate of VOUS (5.6 vs. 5.3%, *p* > 0.05) was equal in early-onset FGR compared with late-onset FGR. The proportion of pathogenic chromosomal anomalies in isolated FGR seemed lower than FGR with ultrasound soft markers (5.3 vs. 9.0%, *p* = 0.528). The incidence of chromosomal anomalies appeared higher in the abnormal fluid group than in the normal fluid group. The incidence of VOUS also increased to 7.1% in the oligohydramnios group. There was one pathogenic CNV in two cases with polyhydramnios. Interestingly, no clinically significant variants were identified when pregnancies were associated with diseases such as gestational hypertension and severe preeclampsia.

**TABLE 1:** Stratified analysis of CNV detection in isolated FGR.

Subgroups	Pathogenic CNVs	Likely pathogenic CNVs	VOUS
GA at the suspicion of FGR			
Early onset (*n* = 195)	16 (8.2%)	1 (0.5%)	11 (5.6%)
Late onset (*n* = 76)	0	1 (1.3%)	4 (5.3%)
*p*-value	0.022	0.483	1.000
Ultrasound soft markers			
Isolated (*n* = 227)	12 (5.3%)	2 (0.9%)	12 (5.3%)
Associated (*n* = 44)	4 (9.0%)	0	2 (4.5%)
*p*-value	0.528	1.000	1.000
Amniotic fluid			
Normal (*n* = 227)	12 (5.3%)	2 (0.9%)	9 (4.0%)
Oligohydramnios (*n* = 42)	3 (7.1%)	0	6 (14.2%)
Polyhydramnios (*n* = 2)	1 (50.0%)	0	0
*p*-value	0.136	0.542	0.020
Type of FGR			
Symmetrical (*n* = 216)	14 (6.5%)	0	11 (5.1%)
Asymmetrical (*n* = 55)	2 (3.6%)	2 (1.1%)	4 (7.2%)
*p*-value	0.632	0.041	0.700
EFW percentile at diagnosis (%)			
<1 (*n* = 234)	16 (6.8%)	2 (0.8%)	13 (5.5%)
≥1 (*n* = 37)	0	0	2 (5.4%)
*p*-value	0.203	1.000	1.000
Umbilical Doppler			
Normal (*n* = 261)	16 (6.1%)	2 (0.8%)	15 (5.7%)
Abnormal (*n* = 10)	0	0	0
*p*-value	0.902	1.000	0.904
Gestational diabetes mellitus			
Normal (*n* = 247)	15 (6.1%)	2 (0.9%)	14 (5.7%)
Associated (*n* = 24)	1 (4.1%)	0	1 (4.1%)
*p*-value	1.000	1.000	1.000
Gestational hypertension			
Normal (*n* = 262)	16 (6.1%)	2 (0.9%)	14 (5.3%)
Associated (*n* = 9)	0	0	1 (11.1%)
*p*-value	0.964	1.000	0.998
Reproductive history			
Nullipara (*n* = 104)	10 (9.6%)	2 (1.9%)	3 (2.8%)
Multipara (*n* = 167)	6 (3.6%)	0	12 (7.2%)
*p*-value	0.041	0.146	0.132
Severe preeclampsia			
Normal (*n* = 255)	16 (6.3%)	2 (0.8%)	13 (5.1%)
Associated (*n* = 16)	0	0	2 (12.5%)
*p*-value	0.627	1.000	0.489

GA, gestational age; CNV, copy number variation; VOUS, variation of uncertain significance.

Table 2 shows the clinical and chromosomal characteristics in 2 cases with aneuploidies and 18 cases with clinically significant variants [pathogenic CNVs (*n* = 16) and likely pathogenic CNVs (*n* = 2)]. Among 18 cases with clinically significant variants, there were 15 cases with CNVs < 10 Mb, but another 3 cases were detected with CNV > 10 Mb. In these 18 cases with CNVs, the type of CNVs in all cases was deletion except one case with duplication. The phenotype involved in these CNVs included 1q21.1 deletion syndrome (*n* = 3), Xp22.31 deletion (*n* = 2), Wolf-Hirschhorn syndrome (*n* = 2), 22q11.21 deletion (*n* = 2), and Williams-Beuren syndrome (*n* = 1). A total of 16 pregnancies in 18 cases chose to terminate the pregnancy after being informed of the abnormal chromosomal tests. However, there were two live births because the time when the two couples were referred to our center from the primary hospital was too late to obtain a CMA result report prenatally. Demographic and chromosomal data of VOUS are shown in Supplementary Table S1. Information on 10 infants was available, showing that two infants were diagnosed with infantile weight below the 3rd percentile but the others were without abnormality.

**TABLE 2:** Clinical characteristics of FGR fetuses with pathogenic/likely pathogenic CNVs.

Case number	GA at the suspicion of FGR	Invasive procedure	Ultrasound soft markers	Amniotic fluid	Microarray results	Type of CNV	Length	Interpretation	Fetal sex	Outcome
1	26 + 4	AC	-	Normal	arr [hg19] 14q32.33 (106251486-106728149)x3	Duplication and deletion	447 kb	Pathogenic	Male/female	TOP
					arr [hg19] Xp22.33q28 (168546-154985852)x1		154.82 Mb			
					arr [hg19] Yp11.31q11.23 (2780527-28799937)x1		26.02 Mb			
2	29 + 1	PUBS	Hyperechogenic bowels	Normal	arr [hg19] 21q11.2q22.3 (15041209_48097372)x3	Duplication	33.06 Mb	Pathogenic	Male	TOP
3	27 + 4	PUBS	-	Normal	arr [hg19] Xp22.31 (6885115_7775073)x1	Deletion	890 kb	Pathogenic	Female	Full-term birth
4	28 + 2	AC	-	Normal	arr [hg19] 1q24.2q31.3 (168061816_198518302)x1	Deletion	30.46 Mb	Pathogenic	Female	TOP
5	23 + 6	PUBS	-	Normal	arr [hg19] 6q26q27 (163579585_170919482)x1	Deletion	7.34 Mb	Pathogenic	Female	TOP
6	24 + 0	AC	-	Normal	arr [hg19] 1q21.1q21.2 (146043713_147897962)x1	Deletion	1.85 Mb	Pathogenic	Male	TOP
7	25 + 0	PUBS	-	Normal	arr [hg19] 20q13.32 (56992676_58241326)x1	Deletion	1.25 Mb	Pathogenic	Male	TOP
8	24 + 0	AC	-	Polyhydramnios	arr [hg19] 22q11.21 (20716877_21800471)x1	Deletion	1.08 Mb	Pathogenic	Female	Preterm birth
9	31 + 6	AC	-	Normal	arr [hg19] Xp22.31 (6455152_8135568)x1	Deletion	1.68 Mb	Pathogenic	Female	TOP
10	26 + 1	AC	-	Normal	arr [hg19] 1q21.1q21.2 (145770679_147897962)x1	Deletion	2.13 Mb	Pathogenic	Male	TOP
11	26 + 6	PUBS	ARSA	Oligohydramnios	arr [hg19] 18p11.32p11.21 (136226_15157836)x3	Duplication	15.02 Mb	Pathogenic	Male	TOP
12	31 + 1	PUBS	-	Oligohydramnios	arr [hg19] Xp22.33 (372012_839488)x1	Deletion	467 Kb	Pathogenic	Female	TOP
13	24 + 1	PUBS	ARSA, PLSVC	Normal	arr [hg19] 4p16.3p16.1 (68345_8731855)x1	Deletion	8.66 Mb	Pathogenic	Female	TOP
14	23 + 0	AC	-	Normal	arr [hg19] 22q11.21 (18916843_20716903)x1	Deletion	1.80 Mb	Pathogenic	Female	TOP
15	24 + 6	PUBS	-	Normal	arr [hg19] 1q21.1q21.2 (146023923_147830830)x1	Deletion	1.81 Mb	Pathogenic	Female	TOP
16	23 + 0	PUBS	Echogenic kidneys	Normal	arr [hg19] 4p16.3p15.2 (68346_22565466)x1	Deletion	22.50 Mb	Pathogenic	Male	TOP
17	29 + 6	PUBS	Mild pericardial effusion	Oligohydramnios	arr [hg19] 13q33.3q34 (109549536_115107733)x1	Deletion	5.56 Mb	Pathogenic	Male	TOP
18	27 + 2	PUBS	-	Normal	arr [hg19] 7q11.23 (72701099_74136633)x1	Deletion	1.44 Mb	Pathogenic	Male	TOP
19	26 + 5	PUBS	-	Normal	arr [hg19] 3q27.1q28 (183265470_192101002)x1	Deletion	8.84 Mb	Likely pathogenic	Male	TOP
20	34 + 0	PUBS	-	Normal	arr [hg19] 6q27 (166870452_170919482)x1	Deletion	4.05 Mb	Likely pathogenic	Female	TOP

GA, gestational age; FGR, fetal grow restriction; CNV, copy number variation; T18, Trisomy 18; T21, Trisomy 21; AC, amniocentesis; PUBS, percutaneous umbilical blood sampling; ARSA, aberrant right subclavicular artery; PLSVC, persistent left superior vena cava; TOP, termination of pregnancy.

Logistic regression showed GA at the suspicion of FGR (OR = 0.846, *p* = 0.014) was the major risk factor for the prediction of chromosomal aberration in fetuses with FGR. Table 3 shows that GA at diagnosis was negatively correlated with the probability of an adverse outcome (OR = 0.902, *p* = 0.008). Increased EFW percentile at the suspicion of FGR was associated with a lower probability of adverse outcomes (OR = 0.504, *p* = 0.014). There was a significant association between asymmetrical FGR and adverse outcomes. We also found that abnormal amniotic fluid and especially severe preeclampsia were risk factors.

**TABLE 3:** Logistics regression analysis in risk factors of adverse outcome in FGR.

Variable	Chromosomal aberration		Adverse pregnancy outcome	
	OR (CI 95%)	*p*	OR (CI 95%)	*p*
Maternal age (years)	0.879 (0.772–1.001)	0.051	1.038 (0.962–1.120)	0.333
EFW percentile at diagnosis (%)	0.637 (0.224–1.812)	0.398	0.504 (0.292–0.871)	0.014
Asymmetrical FGR	0.655 (0.176–2.443)	0.529	2.208 (1.035–4.713)	0.041
GA at diagnosis (weeks)	0.846 (0.741–0.967)	0.014	0.902 (0.837–0.973)	0.008
Ultrasound soft markers	1.891 (0.586–6.099)	0.286	1.855 (0.810–4.245)	0.144
Abnormal amniotic fluid	1.041 (0.303–3.575)	0.950	2.324 (1.041–5.187)	0.040
GDM	1.815 (0.335–9.814)	0.489	0.349 (0.103–1.175)	0.089
Gestational hypertension	N/A	N/A	2.312 (0.307–17.393)	0.415
Abnormal Doppler	N/A	N/A	4.263 (0.380–47.843)	0.240
Severe preeclampsia	N/A	N/A	17.565 (1.971–156.534)	0.010

GDM, gestational diabetes mellitus; OR, odds ratio; CI, confidence interval.

## Discussion

This study confirmed the value of a microarray for genomic unbalanced variants in fetuses with isolated FGR, particularly in fetuses of GA at the suspicion of FGR less than 32 weeks. The CMA had a 6.6% detection rate of clinically significant variants in fetuses with isolated FGR. The detection rate of clinically significant variants was higher in early-onset FGR (< 32 GA weeks) than in late-onset FGR (8.7 vs. 1.3%, *p* < 0.05). Logistic analysis showed early GA at diagnosis of FGR was the major risk factor for chromosomal aberration, showing that the odds of clinically significant variants from CMA in fetuses with isolated FGR decreased by 15.4% when GA at the suspicion of FGR increased by 1 week. Late GA at diagnosis and large EFW percentile of suspicion of FGR were negatively correlated with adverse outcomes for isolated fetuses with FGR. But asymmetrical FGR, abnormal amniotic fluid, and severe preeclampsia could all increase the risk of adverse outcomes.

According to some studies, chromosomal aberration would be more prevalent in early-onset FGR. Zhu et al. (Zhu et al., 2016) have reported that FGR onset in the second trimester without malformation had a higher risk of chromosomal anomalies than those in the third trimester. Peng et al. (Peng et al., 2017) included a total of 128 cases with EFW < 10th percentile and defined a gestational week below 32 as early-onset FGR, finding that chromosomal and sub-chromosomal anomalies occurred significantly more often in early-onset cases. Our data demonstrated 8.7% of chromosomal anomalies in GA below 32 weeks compared with 1.3% of late-onset FGR. The data supported that CMA should be offered in cases of isolated FGR diagnosed before 32 weeks of gestation by the recommendation from the Society for Maternal-Fetal Medicine (SMFM) (Martins et al., 2020). Furthermore, variable regression analysis in our data showed that early GA at diagnosis of FGR was an important risk factor for chromosomal aberration (OR = 0.846), suggesting that the odds of clinically significant variants from CMA in fetuses with isolated FGR decreased by 15.4% when GA at the suspicion of FGR increased every week. To our knowledge, this is the first report to calculate the risk value of genomic unbalanced variants in isolated FGR caused by gestational age at diagnosis.

However, in this study, two fetuses with pathogenic CNVs were born, although both were diagnosed with FGR in the second trimester. As these two infants were 5 and 7 months old at the follow-up time, their results were not revealed via telephone. Long term follow-up is required. Case 3 was diagnosed with heterozygous deletion of Xp22.31, meaning that 50% of male offspring would have X-linked ichthyosis in the future. This is helpful for prenatal counseling. As both infants were diagnosed with FGR in primary hospitals without chromosomal examination resources and the referral time to our center was too late to obtain a CMA report prenatally, the parents were not informed of the findings until after birth. Hence, our findings support the notion that a chromosomal microarray should be recommended in time for FGR despite findings without malformation (Gordijn et al., 2016). Even if patients are diagnosed in a basal hospital, FGR pregnancies should be transferred to a referral center where CMA could be offered as quickly as possible.

Besides gestational ages, we have also explored other potential influencing factors for the detection of chromosomal anomalies in isolated FGR. It was reported that there was an association between severity of FGR and chromosomal aberration rate (Peng et al., 2017), demonstrating that the rate rose from 7.8% in fetuses with EFW below the 10th percentile to 18% for EFW below the 3rd percentile. However, the burden of chromosomal abnormalities for extreme FGR (below the 1st percentile) has been rarely reported. We found that the detection rate of pathogenic and likely pathogenic CNVs increased from 0% with EFW below the 3rd percentile to 7.6% (pCNVs, 6.8% and LP, 0.8%) with extreme EFW (below the 1st percentile). Moreover, some gestational disorders including gestational hypertension and severe preeclampsia were regarded as so-called “definite etiology” leading to FGR. These conditions were probably associated with a lower incidence of chromosomal aberration. Little detection of pathogenic chromosomal abnormalities was observed when pregnancies were associated with gestational hypertension, severe preeclampsia, or abnormal umbilical Doppler in our study. But indeed, it was very difficult to determine if FGR was caused by these disorders or chromosomal abnormalities in clinical practice.

FGR without any malformation could be the only sign in many microdeletions and microduplication syndromes (Monier et al., 2021). Our study found some classical CNVs that several studies have previously reported, including 22q11.2 microdeletion (OMIM: 611867), Xp22.3 microdeletion, and 7q11.23 microdeletion (OMIM: 194050) (Borrell et al., 2017; Monier et al., 2021). Additionally, three cases of isolated FGR with the 1q21.1 microdeletion were observed in our data, and the GA at the suspicion of FGR in these three cases was in the late second trimester. It is widely believed that the 1q21.1 deletion is associated with an unreliable phenotype in the prenatal setting. Many cases with 1q21.1 microdeletion were identified postnatally mainly owing to developmental delay. Few studies reported the association between 1q21.1 microdeletion and isolated fetal growth restriction, probably due to the limited available phenotypes in the prenatal setting (Mefford et al., 2008). In summary, to some degree, our data suggested that 1q21.1 deletion syndrome (OMIM: 612474) should not be overlooked when fetuses are diagnosed with isolated FGR.

Severe fetal growth restriction was associated with increased perinatal morbidity and mortality. We found severe preeclampsia was a major risk factor for perinatal adverse outcomes in isolated FGR. The incidence of stillbirth and perinatal death is significantly increased in severe preeclampsia complicated by fetal growth restriction compared with fetuses without growth restriction (Weiler et al., 2011). Moreover, Dall'Asta et al. recently reported that the EFW percentile at the time of the diagnosis of late-onset FGR was the ultrasound parameter independently associated with adverse perinatal outcomes including prematurity and its potentially related complications in terms of NICU admission, neonatal hypoglycemia, and length of neonatal admission (Dall'Asta et al., 2021). Our data showed that a lower EFW percentile at diagnosis of FGR was a strong predictor for adverse outcomes including intra-uterine fetal death (IUFD), termination of pregnancy (TOP), and preterm birth in pregnancies of FGR.

There are several limitations to our study. Karyotype analysis was not performed for all cases, therefore we probably missed the balanced change of chromosomes. This retrospective study may be limited by recall bias. There was little information on the inheritance of clinically significant variation and VOUS due to the lack of parental CMA data. Additionally, FGR-related long-term phenotypes such as developmental delay and intellectual disability were unlikely observed within the limited follow-up time.

In conclusion, we stress the importance of chromosomal tests for isolated fetal growth restriction and found that early-onset FGR had a higher incidence of chromosomal aberration. The odds of clinically significant variants from CMA in fetuses with isolated FGR decreased by 15.4% when GA at the suspicion of FGR increased every week. The perinatal adverse outcomes were associated with early GA and lower EFW percentile at diagnosis, asymmetry of FGR, abnormal amniotic fluid, and severe preeclampsia.

## Data Availability

The original contributions presented in the study are included in the article/Supplementary Material, further inquiries can be directed to the corresponding author.
