# Supplementary material for: The Genetic and Clinical Outcomes in Fetuses With Isolated Fetal Growth Restriction: A Chinese Single-Center Retrospective Study
Source: Front Genet. 2022 Apr 28;13:856522. doi: 10.3389/fgene.2022.856522 (PMC9096609; doi:10.3389/fgene.2022.856522)
Supplement: Supplementary file 1 [file Table1.docx]

**Supplementary Table 1 Clinical characteristics of FGR fetuses with VOUS**

| **Case number** | **GA at suspicion FGR** | **Invasive procedure** | **Ultrasound soft marker** | **Amniotic fluid** | **Microarray results** | **Type of CNV** | **Length** | **Outcomes** | **Birth weight** | **EFW percentile of birth weight (%)** | **Current age (months)** | **Other**  **abnormality** |  |
| --- | --- | --- | --- | --- | --- | --- | --- | --- | --- | --- | --- | --- | --- |
| 26 | 25+0 | PUBS | - | Oligohydramnios | arr[hg19] 4p15.2p15.1  (27002833_30415930)x1 | Deletion | 3.41Mb | TOP | - |  | - | - |  |
| 29 | 31+5 | PUBS | - | Normal | arr[hg19] 4q26  (116796180_117601048)x3 | Duplication | 805KB | Full-term birth | 2660 | 4.7 | 44 | - |  |
| 31 | 31+3 | PUBS | - | Oligohydramnios | arr[hg19] 11q23.3  (117507945_118575858)x3 | Duplication | 1.07Mb | Preterm | 1350 | 0 | 29 | Current weight less than - 3SD |  |
| 36 | 28+2 | PUBS | - | Oligohydramnios | arr[hg19] Yp11.31q11.221  (2650425_16607014)x3 | Duplication | 13.96Mb | TOP | - | - | - | - |  |
| 37 | 33+2 | AC | - | Normal | arr[hg19] 2q24.1q24.2  (158567198_160680258)x1 | Deletion | 2.11Mb | Full-term birth | 3000 | 14.3 | 34 |  |  |
| 44 | 23+0 | PUBS | - | Normal | arr[hg19] 14q24.2q24.3  (7294293_73813830)x3 | Duplication | 871Kb | Full-term birth | 2700 | 1.2 | 46 | - |  |
| 50 | 32+0 | PUBS | - | Normal | arr[hg19] 10p13  (13986171_14762666)x3 | Duplication | 776Kb | Full-term birth | 2600 | 0.9 | 35 | - |  |
| 52 | 29+0 | AC | - | Oligohydramnios | arr[hg19] 8q21.11q21.12  (77868023_78902320)x3 | Deletion | 1.03Mb | Preterm | 700 | 0 | 5 | Current weight less than - 3SD |  |
| 54 | 24+1 | AC | - | Normal | arr[hg19] 15q11.2  (23288350_23923725)x3 | Duplication | 635Kb | Full-term birth | 2280 | 1.6 | 4 | - |  |
| 58 | 25+3 | PUBS | Hyperechogenic bowels | Normal | arr[hg19] 22q11.1  (1688890_17825432)x3 | Duplication | 937Kb | TOP | - |  | - | - |  |
| 61 | 31+1 | AC | - | Normal | arr[hg19] 14q32.32q32.33  (103384404_105428857)x1 | Deletion | 2.04Mb | Full-term birth | 3100 | 55.6 | 9 | - |  |
| 62 | 33+6 | PUBS | - | Normal | arr[hg19] 18p11.32p11.31  (136226~3367968)x3 | Deletion | 3.23Mb | Full-term birth | 2300 | 0.9 | 47 | - |  |
| 63 | 32+0 | PUBS | - | Oligohydramnios | arr[hg19] 7p22.1  (4905409_5341570)x3 arr[hg19] 12p13.33  (173786_365726)x3 | Duplication Duplication | 192Kb 192Kb | LFU | - | - | - | - |  |
| 64 | 24+2 | PUBS | - | Oligohydramnios | arr[hg19] 5q23.3  (129135230_129753265)x1 | Deletion | 618Kb | Full-term birth | 2500 | 0.1 | 14 | - |  |
| 65 | 30+0 | AC | Hyperechogenic bowels | Normal | arr[hg19] 2p25.3  (970388_1744932)x3 | Duplication | 775Kb | LFU | - | - | - | - |  |
| GA, gestational age; FGR, fetal grow restriction; CNV, copy number variation; AC, amniocentesis; PUBS, percutaneous umbilical blood sampling; PLSVC, persistent left superior vena cava; TOP, termination of pregnancy; VOUS, variation of uncertain significance; LFU, lost to follow up; EFW, estimated fetal weight. | | | | | | | | | | | | |  |
